# Efficacy of the combination of modern medicine and traditional Chinese medicine in pulmonary fibrosis arising as a sequelae in convalescent COVID-19 patients: a randomized multicenter trial

**DOI:** 10.1186/s40249-021-00813-8

**Published:** 2021-03-18

**Authors:** Zhen-Hui Lu, Chun-Li Yang, Gai-Ge Yang, Wen-Xu Pan, Li-Guang Tian, Jin-Xin Zheng, Shan Lv, Shao-Yan Zhang, Pei-Yong Zheng, Shun-Xian Zhang

**Affiliations:** 1 Longhua Hospital, Shanghai University of Traditional Chinese Medicine, Shangha, 200032 People’s Republic of China; 2 The 903Rd Hospital of People’s Liberation Army of China, Hangzhou, 310013 People’s Republic of China; 3 Guangzhou Women and Children’s Medical Center, Guangzhou, 510623 People’s Republic of China; 4 The First Affiliated Hospital of Jinan University, Jinan University, Guangzhou, 510632 People’s Republic of China; 5 National Institute of Parasitic DiseasesChinese Center for Disease Control and PreventionChinese Center for Tropical Diseases ResearchKey Laboratory of Parasite and Vector BiologyMinistry of HealthNational Center for International Research On Tropical DiseasesMinistry of Science and Technology, WHO Collaborating Center for Tropical Diseases, Shanghai, 200025 People’s Republic of China; 6 School of Global Health, Chinese Center for Tropical Diseases Research-Shanghai Jiao Tong University School of Medicine, Shanghai, 200025 People’s Republic of China

**Keywords:** COVID-19, SASR-CoV-2, Pulmonary fibrosis, Modern medicine, Traditional Chinese medicine

## Abstract

**Background:**

The coronavirus disease 2019 (COVID-19) caused by severe acute respiratory syndrome coronavirus-2 (SARS-CoV-2) has led to a significant number of mortalities worldwide. COVID-19 poses a serious threat to human life. The clinical manifestations of COVID-19 are diverse and severe and 20% of infected patients are reported to be in a critical condition. A loss in lung function and pulmonary fibrosis are the main manifestations of patients with the severe form of the disease. The lung function is affected, even after recovery, thereby greatly affecting the psychology and well-being of patients, and significantly reducing their quality of life.

**Methods:**

Participants must meet the following simultaneous inclusion criteria: over 18 years of age, should have recovered from severe or critical COVID-19 cases, should exhibit pulmonary fibrosis after recovery, and should exhibit Qi-Yin deficiency syndrome as indicated in the system of traditional Chinese medicine (TCM). The eligible candidates will be randomized into treatment or control groups. The treatment group will receive modern medicine (pirfenidone) plus TCM whereas the control group will be administered modern medicine plus TCM placebo. The lung function index will be continuously surveyed and recorded. By comparing the treatment effect between the two groups, the study intend to explore whether TCM can improve the effectiveness of modern medicine in patients with pulmonary fibrosis arising as a sequelae after SARS-CoV-2 infection.

**Discussion:**

Pulmonary fibrosis is one of fatal sequelae for some severe or critical COVID-19 cases, some studies reveal that pirfenidone lead to a delay in the decline of forced expiratory vital capacity, thereby reducing the mortality partly. Additionally, although TCM has been proven to be efficacious in treating pulmonary fibrosis, its role in treating pulmonary fibrosis related COVID-19 has not been explored. Hence, a multicenter, parallel-group, randomized controlled, interventional, prospective clinical trial has been designed and will be conducted to determine if a new comprehensive treatment for pulmonary fibrosis related to COVID-19 is feasible and if it can improve the quality of life of patients.

*Trial registration:* This multicenter, parallel-group, randomized controlled, interventional, prospective trial was registered at the Chinese Clinical Trial Registry (ChiCTR2000033284) on 26th May 2020 (prospective registered). 
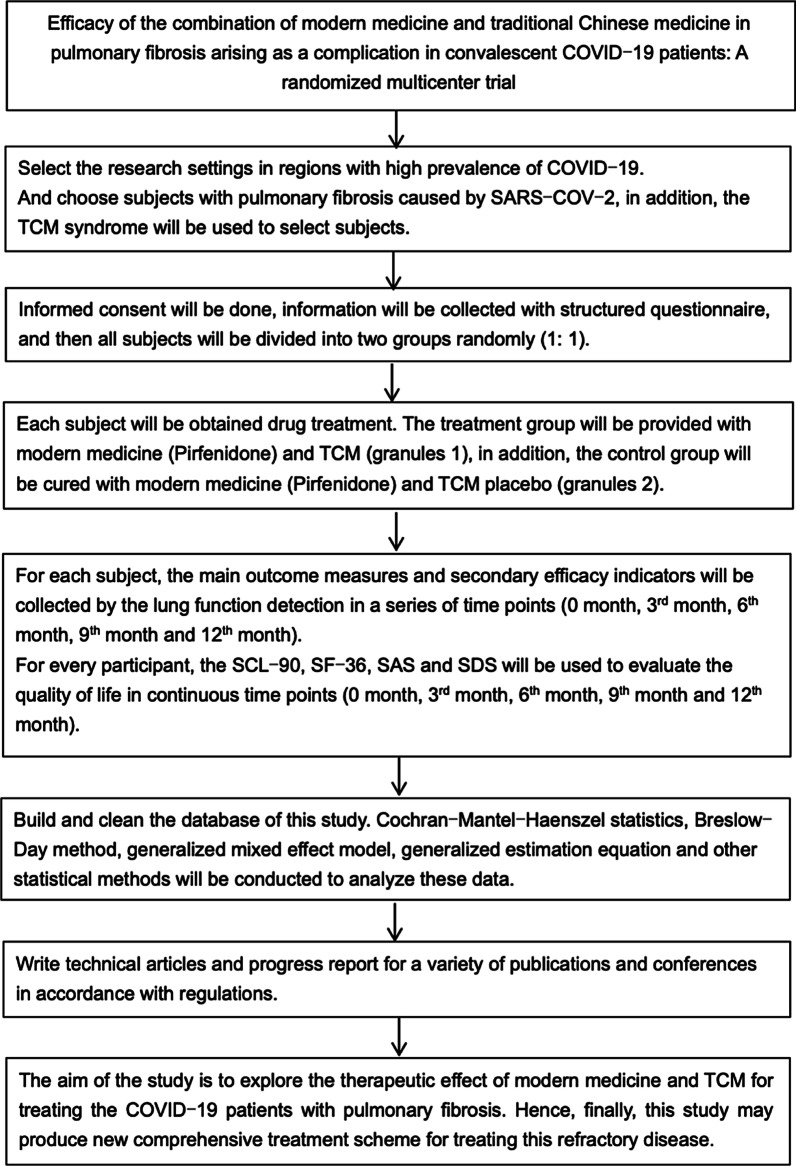

## Background

Coronavirus disease-2019 (COVID-19) was discovered in December 2019, it has resulted in a sudden and significant number of hospitalizations and pneumonia-related deaths globally to date [1–3]. COVID-19 is associated with morbidity and mortality globally and has been defined as an “international emergency” and characterized as a pandemic by the World Health Organization (WHO) [1]. Currently, this infection is prevalent in more than 215 countries. As of February 21, 2021, over 111.07 million confirmed cases and 2.46 million deaths resulting from COVID-19 have been reported globally [4]. The rapid spread of COVID-19 poses significant economic, social, and healthcare challenges worldwide.

The causative organism of COVID-19 is the severe acute respiratory syndrome coronavirus-2 (SARS-CoV-2) [1, 2, 5]. Epidemiological data suggests that droplet transmission is the most common transmission route and that surface contact is another important mode of transmission [6]. Patients are the most important source of transmitting infection [7, 8]. Additionally, asymptomatic patients may play a role in transmission and should not be neglected [9].

COVID-19 can lead to a wide spectrum of respiratory diseases with an extremely high incidence of acute respiratory distress syndrome. It has various clinical manifestations [9], ranging from asymptomatic infections to mild respiratory tract disease and severe interstitial pneumonia, which may lead to respiratory failure and death [6]. About 81% of patients with COVID-19 exhibit mild symptoms [7], characterized by fever, fatigue, and dry cough. A few patients with mild infection show signs of nasal congestion, runny nose, sore throat, and diarrhea [7], whereas none of the mild cases exhibit pulmonary inflammation upon imaging. Patients with the mild form of the infection recover completely within 1–2 weeks. However, 19% of patients with COVID-19 exhibit severe manifestations [7], including respiratory failure, septic shock, and multiple-organ dysfunction, it is characterized by progressive dyspnea and intractable hypoxemia. These conditions are difficult to treat and can prove to be fatal in the absence of prompt and specific medical treatment [7].

Currently, there are no specific or effective options (including remdesivir, convalescent and plasma therapy) to treat severe or critical COVID-19 cases [11–13], some comprehensive treatment methods were used to treatment those fatal cases fact to experience principle, the therapy methods involved using antivirals, antibiotics, hormones, and mechanical ventilation [14–16]. Although most patients can be cured, a small number of patients may exhibit lung injury after recovery [17] and pulmonary fibrosis is one of the sequelae of COVID-19 [18, 19]. This progressive disease is characterized by a significant decrease in lung function, which cause respiratory failure and death. The common clinical manifestations of pulmonary fibrosis are dyspnea and deterioration of the lung function index. Currently, lung transplantation is the only viable treatment option that is known to improve outcomes [20]. Moreover, the recovery of pulmonary function is a slow process [21, 22] and patients may experience several drug-induced side effects [21, 22]. Hence, both the mental and physical functions of patients are greatly affected, along with a considerable decline in the quality of life. The burden of fibrotic lung disease following SARS-CoV-2 infection is likely to be high, and the global burden is likely to further increase, considering the steady rise in the number of individuals afflicted with COVID-19 [21].

There is evidence that drugs, such as pirfenidone and nintedanib, can delay the decline of lung function in patients with pulmonary fibrosis [23]. However, the natural course of pulmonary fibrosis is irreversible. Drug therapy using antifibrotic agents may be beneficial in the treatment of COVID-19 through a range of mechanisms, and prevent long-term adverse consequences. Traditional Chinese medicine (TCM) has been commonly used in the prevention of fibrosis and improvement of lung function [24–26].

**Fig. 1:**
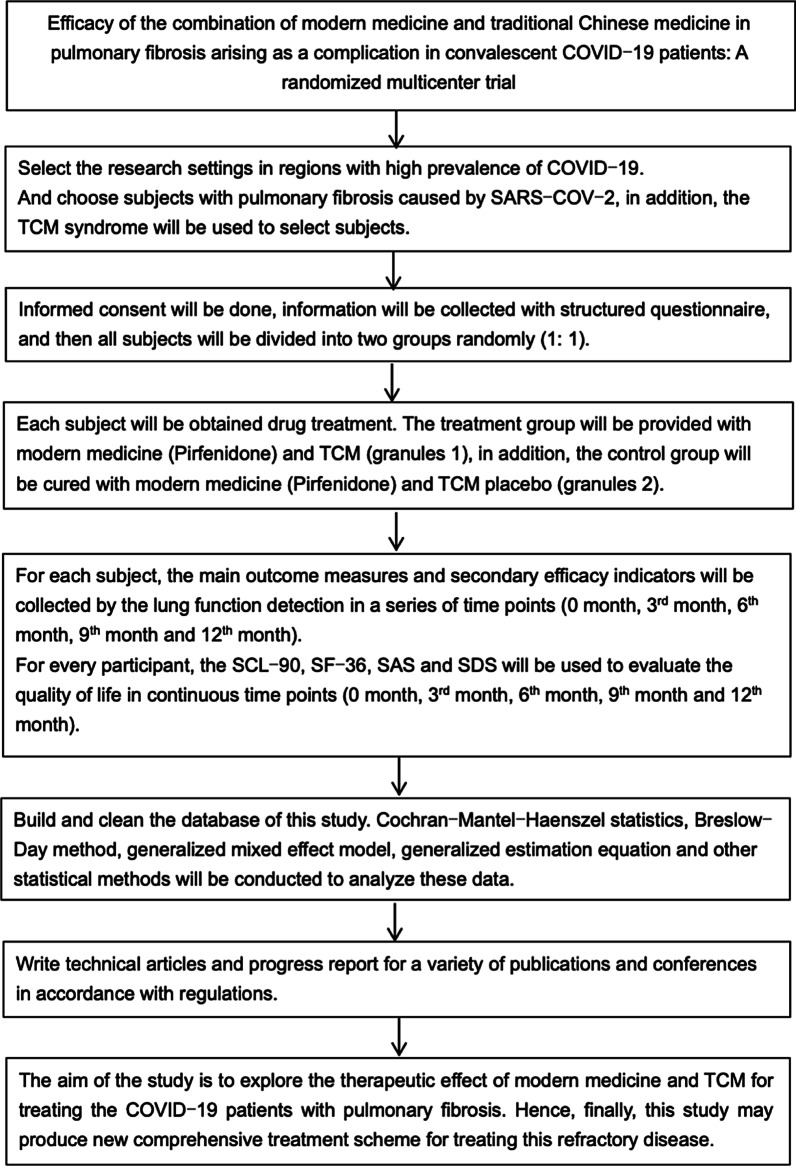
The technology roadmap of this study. *COVID-19* Corona Virus Disease 2019, *SARS-CoV-2* Severe acute respiratory syndrome coronavirus 2, *SAS* Self-rating anxiety scale, *SCL-90* Symptom self-evaluation scale; *SDS* Self-rating depression scale, *SF-36* 36-Item short form health survey, *TCM* Traditional Chinese medicine;

Parallel, double-blind, multicenter, randomized clinical trials (RCTs) constitute a reliable method for evaluating drug efficacy. It is hypothesized that the integration of TCM and modern medicine may be effective in the treatment of pulmonary fibrosis caused by COVID-19. Based on this combination, a new treatment for pulmonary fibrosis may be obtained, it may significantly improve the life expectancy of patients with pulmonary fibrosis caused by COVID-19 (Fig. 1).

### Aim of the study

This multicenter, parallel, double-blind RCT will be conducted in a clinical setting. Patients who recovered from severe and critical COVID-19 and subsequently developed pulmonary fibrosis will be recruited for this study. Additionally, all subjects will have Qi-Yin deficiency TCM syndrome. The enrolled subjects will be divided randomly into the treatment or control group. Subjects in the treatment group will receive modern medicine plus TCM, whereas those in the control group will be provided modern medicine plus a TCM placebo. The lung function index and quality of life score will be continuously examined for each participant. By comparing the treatment effect between the two groups, we will explore whether TCM can improve the effectiveness of modern medicine when used to treat pulmonary fibrosis induced by COVID-19. There are several outcomes that we will evaluate from this study. First, we will discover the basic characteristics of pulmonary fibrosis related to COVID-19. Second, we will explore the risk factors of pulmonary fibrosis-related COVID-19 that could provide references for the identification of COVID-19. Third, we will determine the influencing factors that could improve the outcomes of pulmonary fibrosis-related COVID-19. Fourth, we will compare the efficacy of modern medicine + TCM versus modern medicine + TCM placebo, it can help screen and optimize a novel comprehensive treatment approach for effectively improving the outcome and quality of life of patients with induced by COVID-19.

### Hypothesis

Some studies have reported that TCM can significantly reduce the severity of pulmonary fibrosis induced by several factors, including pathogens [27, 28]. The efficacy of TCM in improving lung function has also been established partly [27, 28]. In addition, some studies have found that the combination of modern medicine and TCM is effective in improving lung function and the quality of life of patients compared to therapy using modern medicine and/or TCM placebo [29, 30]. Hence, it was hypothesized that Using modern medicine (Pirfenidone) with TCM may promote lung function in subjects who recovered from severe or critical COVID-19. Thus, the study will likely provide novel insights into the long-term outcomes of COVID-19 in this category of patients.

### Research methods/design

#### Ethical aspects of the study

This study has been approved by the Chinese Clinical Trial Registry (ChiCTR2000033284) and the ethical review committee of Guangzhou Women and Children’s Medical Center Research Ethics Committee (No.202028500). All experimental procedures will be performed in accordance with the declaration of Helsinki and written informed consent will be obtained from all participants following a detailed description of the purpose, potential risks, and benefits of the study. Participation will be voluntary and will be offered the choice to withdraw from the study at any time without any obligations.

##### Study setting

The study will be carried out in provinces where the prevalence of COVID-19 was high, thereby comprising a large pool of patients with severe and critical COVID-19. Several hospitals will be selected, as it is likely that several patients with lung function damage (including pulmonary fibrosis) related to COVID-19 will be enrolled. In addition, only physicians in these hospitals who are experienced in the treatment of respiratory diseases will be recruited. Moreover, these physicians will be trained in the aspects of the theory and principle of TCM, such that the research team can function collaboratively and seamlessly.

##### Sample size calculation

For dichotomous variable and superior clinical design, the sample size was determined using the following formula to calculate the sample size [29, 30, 31]:

$$N = 2 \times \left( {\frac{{Z_{1 - \alpha } + Z_{1 - \beta } }}{{d - \delta_{0} }}} \right)^{2} \times p \times (1 - p)$$,

where the null hypothesis H_0_: T–S = δ;

the alternative hypothesis H_1_: T–S > δ;

And test statistics: Z = (d–δ)/sd.

In the equation, N represents the size of the group. p indicates the response rate of the control group and p_0_ denotes the response rate of the treatment group. Z_x_ represents the normal standard deviation for a one- or two-sided x. d represents the real difference between the two treatment effects. δ_0_ indicates a clinically acceptable margin. S^2^ indicates the pooled standard deviation of both comparison groups. T represents the treatment group (modern medicine + TCM treatment). S is the control group (modern medicine + TCM placebo). δ indicates the clinically admissible margin of superiority. d represents the effectiveness difference between T and S (d =| p_0_—p |). sd represents the standard error of d. Z = Z obeys the normal standard distribution. α represents type I error, β represents type II error, and 1 − β indicates the power.

In this study, α = 0.05, β = 0.20, δ_0_ = 0.10, 1 − β = 0.80, Z_1−α_ = 1.64, Z_1−α_ = 1.28, and based on literature, *P*_0_ = 0.767 and *P* = 0.533[29]. Hence, a value of *N* = 233 was calculated. The failure to follow-up rate was considered to be 10% and accordingly, *N* = 233 × (1 + 10%) = 257. Lastly, the number of subjects each in the treatment and control groups is 257, which led to a total of 514 individuals in this study.

##### Inclusion criteria

Patients who meet all the following inclusion criteria may be included in the clinical trial:

1. Diagnostic criteria for severe or critical COVID-19
  Patients exhibiting positive etiological evidence of the SARS-CoV-2 nucleic acid determined using real-time polymerase chain reaction (PCR) or gene sequencing will be included. In addition, patients should meet at least one of the following diagnostic criteria of severe COVID-19. First, shortness of breath and a respiratory rate (RR) ≥ 30 times/min. Second, oxygen saturation (SpO_2_) in quiescent condition ≤ 93% using finger measurement. Third, arterial partial pressure of oxygen (PaO_2_)/oxygen inhalation concentration (FiO_2_) ≤ 300 mmHg (1 mmHg = 0.133 kPa). The PaO_2_/FiO_2_ will be recalculated when patients will be enrolled from regions at high altitudes, especially those located 1000 m above sea level, using the following formula: PaO_2_/FiO_2_ × [atmospheric pressure (mmHg) /760].
  The diagnostic criteria of critical COVID-19 should meet one of the following criteria, including respiratory failure and those who needed mechanical ventilation, shock, or respiratory failure combined with organ failure, which required monitoring and treatment in an intensive care unit (ICU).
2. Diagnostic criteria for pulmonary fibrosis
  The diagnosis criteria of pulmonary fibrosis will be in accordance with the guidelines of evidence-based medicine (2011 Edition) of the ATS, European Respiratory Society (ERS), Japanese Respiratory Society (JRS), and the Latin American Thoracic Association (ALAT).
  The inclusion criteria for patients with pulmonary fibrosis should meet one of the following conditions. First, subjects should present diffuse, asymmetric reticular formation in the lungs. Second, the reticular formation should show nodular shadows in a lung X-ray and there should be reticular shadows in the lower lobes of both lungs, especially under the pleura. Third, subjects should exhibit restrictive ventilation dysfunction, decreased lung volume, functional residual volume, and normal or increased forced lung volume (FLV)/ forced expiratory volume in 1 s(FEV1) upon assessing lung function. In addition, they should exhibit decreased carbon monoxide(CO) levels exhaled in the single-breath determination of CO. Open chest or thoracoscopic lung biopsy will be considered the “gold standard" for the diagnosis of pulmonary fibrosis.
  In this study, the pulmonary function indices must meet the following criteria. First, the predicted forced vital capacity (FVC%) must be between 50 and 90%. Second, the diffusion capacity for carbon monoxide (DLCO) should be between 30 and 90%. Third, the FEV1/forced vital capacity(FVC) must be over 80%. Fourth, when subject is at rest in a room and is respiring normally, the oxygen partial pressure should more than 50 mmHg.
3. TCM syndrome differentiation for subjects
  The criterion of TCM syndrome differentiation complies with TCM disease diagnosis and efficacy standards promulgated by the China State Administration [32]. In addition, the criterion of Qi-Yin deficiency must meet the following criteria, including shortness of breath, cough, sputum expectoration, a small amount of yellow and white sputum, spontaneous sweating, night sweats, fatigue, drawl, dry mouth, afternoon hot flashes, hot facial flashes, anorexia, red tongue, thin white coating on the tongue, and weak pulse[32]. Qi-Yin deficiency was found to account for 41.5% (39/94) of all individuals with pulmonary fibrosis in other studies[33].
4. Age: ≥ 18 years
5. Written informed consent must be obtained from the patient or from their legal guardian. Patient may also be enrolled upon confirmation of the urgency of participation in the clinical trial and the possible benefit to the patient assessed by an independent consultant, or the implementation of other established procedures to include patients who are unable to provide informed consent.

##### Exclusion criteria

Patients who meet any of the following exclusion criteria will be excluded from this trial. First, diagnosis of any lung diseases, such as bronchiectasis, tuberculosis, bronchial asthma, chronic obstructive pulmonary disease (COPD), dyspnea, respiratory failure, lung cancer, or those with a history of these diseases. Second, patients with a status of acute exacerbation of pulmonary fibrosis. Third, patients with severe disorders of the cardiovascular system, nervous system, and digestive system; serious mental diseases, tumors, human immunodeficiency virus (HIV) infection/acquired immune deficiency syndrome (AIDS), tuberculosis, autoimmune diseases, and severe liver and kidney dysfunction. Fourth, patients who received glucocorticoids, immunosuppressants, and other drugs for treatment of pulmonary fibrosis or other diseases in the past 3 months. Fifth, participation in another interventional clinical trial within the past 3 months. Sixth, patients who did not provide informed consent. Finally, lactating women will be excluded from the study.

### Randomization and blinding

The double-blind method will be used for all aspects of this study including the enrolment of participants, investigators, care providers, outcome assessors, and data analysts. A statistician labelled and distributed the study drugs to each site. A study coordinator will be blinded and participants will be assigned in a 1:1 ratio to treatment groups based on the SAS Analytics Software Solutions (version 9.4, SAS Institute Inc., Cary, North Carolina 27,513, US), it will be used to generate random codes using the stratified randomization method. The study center will be the stratification factor. Pharmacists at the pharmacies of all sites of the clinical trial will be blinded to the patient characteristics and will distribute drugs to the patients according to the instructions of the investigators. The selected block length and random initial value seed parameters will be considered confidential data.

#### Research contents

The lung function index, blood oxygen saturation, and quality of life will be continuously monitored and recorded for each subject (Fig. 1).

Lung function parameters will be measured are as follows, including vital capacity (VC), total vital capacity (TLC), FVC, FLV, FVC%, DLCO, and predicted diffusion capacity for carbon monoxide (DLCO%). Furthermore, FEV1, residual volume (RV), maximum vital capacity (MVV), expiratory reserve volume (ERV), inspiratory reserve volume (IRV), peak expiratory flow (PEF), maximal middle expiratory flow (MMEF), tide volume (VT), and inspiratory capacity (IC), will be also measured, among others.

Detection of blood oxygen saturation will be determined through arterial blood oxygen saturation (SaO_2_) and partial pressure of oxygen (PaO_2_), among other indicators measured using a pulse oximeter.

A 6-min walking distance test will be conducted to assess lung function according to the American Thoracic Society (ATS) criterion.

#### Dyspnea assessment: elevated Borg dyspnea score

SGRQ: SGRQ will be used to evaluate the impact of chronic airflow limitation on the quality of life. The questionnaire contains 50 items, including symptoms, activity ability, and the impact of the disease on daily life, which will be scored over a range of 0–100 points. The higher the score, the worse the health status of patients.

#### Quality of life assessment

SF-36 table, SCL-90 table, SAS table, and SDS table will be constructed to assess the quality of life.

#### Investigational drug

##### Therapeutic schedule

In this study, TCM experts will identify and select the research subjects with Qi-Yin deficiency based on TCM syndrome differentiation, while experts in modern medicine will formulate the drug prescription and design a treatment plan. All subjects will be randomly divided into a treatment or control group in a 1:1 ratio. The treatment group will receive modern medicine + TCM herb granule 1, while the control group will be treated using modern medicine + TCM herb granules (placebo). During treatment, the dosage of the TCM herb or pirfenidone was not adjusted for any subject.

##### Drug preparation and treatment program

The treatment of pulmonary fibrosis will be in adherence to "The 2018 Diagnosis of Idiopathic Pulmonary Fibrosis Guidelines" as symptomatic treatment is indispensable for patients with pulmonary fibrosis [34–36]. Drugs including pirfenidone, acetylcysteine, and glucocorticoids, which have been proven to be efficacious in the treatment of pulmonary fibrosis will constitute the modern drugs in this study.

TCM herb granule 1 will be will be formulated as mixed granules for single TCM herb administration and packed in food-grade plastic bags (8 g/bag), the granules will comprise extracts of adenophora tetraphylla, radix glehniae, liriope graminifolia, codonopsis pilosula, selfheal, meretricis, angelica sinensis, arrowhead mushroom, oysters, seaweed, peach kernel, the root of red-rooted salvia, curcuma aromatica, rhizome sparganii, radices zedoariae, and radix astragali. TCM herb granules 2 is placebo and will be formulated using supplementary materials, and it will be packed in food-grade plastic bags (8 g/bag) (Table 1). The method of use of each drug is shown in Table 1.

**Table 1:** Therapeutic regimen of this study

Drug	Dosage	Taking drug	Remarks
Pirfenidone	The initial dosage is 200 mg/time, 3 times/day; and then add the dose to 600 mg/time by 200 mg/day. Finally, the dose is 600 mg/time, 3 times/day for 12 months	Take it half an hour after breakfast, lunch and dinner, respectively	Drug must be used for each participant
Acetylcysteine	200 mg/time, 3 times/day for 12 months	Take it half an hour after breakfast, lunch and dinner, respectively	Selective use for all subjects
Glucocorticoid	0.5 mg × kg^−1^ × day^−1^ for 1 month, and then 0.25 mg × kg^−1^ × day^−1^ for 2 months, and then the dose decline to 0.125 mg × kg^−1^ × day^−1^ for 12 months	Take it half an hour after breakfast, lunch and dinner, respectively	Selective use for all subjects
Chinese medicine granules 1	8 g/bag, 2 bags/ time, 3 times/day for 12 months	Take this granula after mixing it with water, take it half an hour after breakfast, lunch and dinner, respectively	Drug must be used for each individual in treatment group
Chinese medicine granules 2	8 g/bag, 2 bags/time, 3 times/day for 12 months	Take this granula after mixing it with water, take it half an hour after breakfast, lunch and dinner, respectively	Drug must be used for each volunteer in control group

*kg* kilogram, *mg* milligram

#### Pharmaceutical administration

##### Drug delivery of modern medicine

Each subject will be administered modern medicine (pirfenidone, acetylcysteine, or glucocorticoid), irrespective of the group they have been assigned to.

##### Dispensing the herbal medication

Each center will have a drug administrator who is responsible for the storage, distribution, recovery, record keeping, and retrieval of the experimental drugs. The modern medicine + TCM or modern medicine + TCM placebo will be provided to patients based on the enrollment sequence. The modern medicine + TCM or modern medicine + TCM placebo administered to patients will remain unchanged throughout the study. The test drugs will be distributed in each follow-up after the beginning of the study, and the leftover drugs will be recovered in the next drug allocation.

##### Subject compliance and drug inventory

During the trial, patient compliance will mainly be centered around ensuring drug intake based on the advice of the physician or the drug administrator. Measures will be taken to ascertain that each subject adequately understands the importance of medicating. They will also be educated to ensure that the dosage instructions are appropriately followed and they prevent the concurrent use of other drugs or treatment methods during the study period.

The physician in charge will be trained to count and recover the remaining drugs from patients during follow-up and will be educated to ask questions on whether the subject has taken the prescribed dose of the medication. The attending physician will be trained to record information in cases of missed or insufficient doses and present the information as a case report form (CRF), which will be used to determine patient compliance.

### Outcomes

#### Primary outcome

The main outcome measure is the treatment effect, which will measure the classification indicators, such as improvement, stabilization, and deterioration levels. This three-level classification will monitor changes in TLC, VC, DLCO, SaO_2,_ and PaO_2._ The specific classification criteria will be as follows:

Improvement: after treatment of subjects, an increase in the TLC or VC by 10%, an increase in DLCO by 15%, an increase in SaO_2_ by 15%, and an increase in PaO_2_ by 4 mm Hg (1 mmHg = 0.133 kPa), compared to the pre-treatment values.

Stabilization: the health status of subjects between improvement and deterioration.

Deterioration: after treatment of subjects, a decrease in the TLC or VC by 10%, a decrease in DLCO and SaO_2_ by 15%, and a decrease in PaO_2_ by 4 mm Hg (1 mmHg = 0.133 kPa), compared to the pre-treatment values.

#### Secondary outcome measure

- *(1) Indices reflecting respiratory function*

Certain variables will be monitored for each subject during every follow-up, including VC, TLC, FVC, FVC%, DLCO, DLCO%, FEV1, RV, MVV, RV/TCL, ERV, IRV, PEF, MMEF, VT, and IC.

- *(2) Six-minute walking distance and Borg dyspnea score*

The quantitative variable of 6-min walking distance and the Borg dyspnea score will be obtained for each subject in each observation.

- *(3) Quality of life assessment indices*

The SCL-90, SF-36, SAS, and SDS scales will be used to collect the score (quantitative variable) for each subject during each visit.

- *(4) Evaluation standard of treatment effect in TCM syndrome*

The treatment effect in TCM syndrome will be divided into four categories based on the guiding principles for clinical research of the new TCM (Trial) (2018 year). The categorical variables are clinical cure, notable effect, effective, and ineffective. The specific classification criteria are as follows:

Clinical cure: a reduction rate of TCM syndrome scores ≥ 95%;

Notable effect: a reduction rate of TCM syndrome scores to between 70 and 95%.

Effective: a reduction rate of TCM syndrome scores to between 30 and 70%.

Ineffective: a reduction rate of TCM syndrome scores < 30%.

The reduction rate of TCM syndrome scores(%) = (pre-treatment TCM syndrome scores-post-treatment TCM syndrome scores) / TCM syndrome scores × 100%

The total effective rate(%) = (clinical cure cases + notable effect cases + effective cases) / total cases × 100%.

The data will be analyzed to determine if there are significant differences between the treatment and control groups after treatment, if there are significant differences in the treatment group pre- and post-treatment, and if there are significant differences in the control group pre- and post-treatment.

#### Safety indices

It is necessary to pay close attention to any changes in the relevant safety indicators during treatment as certain therapeutic drugs (especially herbs) may cause organ damage. The safety indices include body temperature, blood pressure, heart rate, respiration, height, and weight, among others[37] (Table 2). Upon and after recruitment, the safety indices of all subjects will be determined several times during the 12-month treatment procedure.

**Table 2:** Safety indexes will be detected for each volunteer in the study

Classification	Index	Detection time point
Blood routine	Red blood cell, hemoglobin, white blood cell, white blood cell classification count, hematocrit, platelet, etc	When one volunteer will be recruited, these safe indexes will be detected for five times (0 month, 3rd month, 6th month, 9th month, 12th month) across whole study
Urine routine	PH, urine specific gravity, urobilinogen, urine blood, white blood cell, urine protein, glucose in urine, bilirubin, ketone body, urine red blood cell, etc	
Liver function	Total protein, globulin, albumin, alanine aminotransferase, aspartate aminotransferase, total bilirubin, direct bilirubin, indirect bilirubin, urine bilirubin, urobilinogen, serum bile acid, gamma glutamyltranspeptidase, alkaline phosphatase, etc	
Kidney function	Serum urea, uric acid, blood β-microglobulin, serum creatinine, etc	
Blood sugar	Fasting blood sugar	
Electrocardiogram	Chest leads, limb lead	

*PH* hydrogen ion concentration

### Participant withdrawal

Participation in this clinical trial is voluntary. All subjects have the right to withdraw their consent prematurely from the trial, at any time and without stating reasons, and without incurring any setbacks for their future medical treatment. Withdrawal of consent will automatically lead to the disqualification of the patient. No further study-related measure will be carried out. However, the stored data may continue to be used, as deemed necessary, to determine the effects of the intervention.

### Suspension of protocol

Participation of a patient in the clinical trial may be prematurely terminated under the following circumstances: (1) death of the participant, (2) failure to obtain consent to the continuation of the clinical trial, and (3) refusal to continue the clinical trial by formerly incapacitated patients who have regained their ability to provide consent.

The principal investigator have the power to pause or terminate the clinical trial in a trial center in coordination with the Safety Committee and the biometrician for a number of reasons. These include if (1) serious safety problems occur during the study period. (2) The drug efficacy is found to be poor or ineffective midway during the study. 3) Major errors or flaws are detected in the trial design. (4) The research team requests the termination of the study for reasons including, but not limited to, funding, management, or a lack of study subjects for recruitment. (5) The administrative department cancels the study.

### Data management and monitoring

#### Principle

Experimental data will be carefully entered into an electronic CRF. To guarantee data quality, we will appoint a research assistant who will be responsible for quality control during data collection. Participant identity and sensitive information will be deleted from all study documents to protect patient confidentiality. All outcomes will be double-checked manually and a second independent data entry will be conducted to promote data quality. Following data entry, only the personnel directly involved in data analysis will be granted access to the final trial datasets. During the study, an independent data and safety monitoring board of the Global Health Center of Shanghai Jiao Tong University will be responsible for monitoring the safety, progress, study integrity, and design aspects of the trial. Dropouts and withdrawals will be recorded throughout the intervention and the data will be collected in the CRF and used for final statistical analysis. No conflicts of interest with the sponsors or researchers exist.

#### Collection of information after volunteers are enrolled in this study

Some general and basic information will be collected using a structured questionnaire upon patient enrolment, including weight, height, age, gender, birthplace, residence, education level, religious beliefs, and income levels. In addition, the clinical manifestations of every individual will be collected, including fatigue, night sweats, chest tightness, chest pain, dyspnea, insomnia, weight loss, and loss of appetite. The structured questionnaire will also be used to collect information that may considerably affect treatment efficacy and outcomes. These factors include smoking and alcohol consumption, socio-economic patterns such as floating population and tramp, working conditions (such as exposure to dust, high temperature, and high humidity), and conditions affecting the lungs (silicosis, pneumoconiosis, tuberculosis). Other factors include the use of antibiotics, and debilitating conditions such as HIV/AIDS, hepatitis B, COPD, hyperthyroidism, hypertension, nervous system diseases (disorders, abnormalities), hyperuricemia, cardiovascular diseases (including coronary heart disease), lung cancer, and tumors, among others.

The treatment information for COVID-19 will also constitute a key influencing factor to explore the efficacy of the treatment in this study. The key information will include weight (before and after treatment), SARS-CoV-2 infection time, diagnosis time, length of the hospital stay, and pulmonary infections caused by other pathogens. Furthermore, the use of antibiotics, plasma therapy, alpha interferon, lopinavir/ritonavir, ribavirin, nasal catheter oxygen inhalation, mask oxygen inhalation, invasive mechanical ventilation, extracorporeal membrane oxygenation (ECMO), immunosuppressants, corticosteroids, and TCM, we also recorded.

#### Information collection from participants during each follow-up

Each subject enrolled in the study will be followed-up once every 3 months and the described indices will be determined each time. A total of 5 (months 0, 3, 6, 9, and 12) determinations per subject will be made during the study period.

In each follow-up, a structured questionnaire will be used to collect clinical information and indices. These included cough, expectoration, fatigue, chest tightness, chest pain, dyspnea, insomnia, emaciation, loss of appetite, VC, TLC, FVC, FVC%, DLCO, DLCO%, FEV1, RV, MVV, RV/TCL, ERV, IRV, PEF, MMEF, VT, IC, 6-min walking distance, Borg dyspnea score, and TCM syndrome scores, which are reflective of respiratory function and will help determine TCM syndrome scores. Moreover, the SCL-90, SF-36, SAS, and SDS scales will be used to record and measure quality of life for each individual during every follow-up.

### Statistical analysis

#### Database

The database will be generated using EpiData 3.1 (The EpiData Association, Odense, Denmark) and all data will be recorded by two operators and tested for consistency. Three databases will be established, including a full analysis set (FAS), per protocol set (PPS), and safety set (SS). The total number of selected and completed cases in each study center will be shown and the number of dropouts and the reasons will also be listed.

#### Principles of data analysis

Statistical analysis will be performed using the SAS Analytics Software Solutions (version 9.4, SAS Institute Inc., Cary, North Carolina 27,513, US). Odds ratio (OR) and 95% confidence interval (*CI*) of categorical variables will be calculated using two-tailed Chi-square test, and Fisher’s exact test will be used to compare the differences in qualitative variables between the two groups. Quantitative variables will be described as the mean, median, standard deviation, or inter-quartile range, and analyzed using the rank-sum test, t-test, analysis of variance (ANOVA), repeated measurement ANOVA, generalized linear mixed model (GLMM), and generalized estimation equation (GEE). Differences will be considered significant at *P* < 0.05 with two-tailed test.

This study will be conducted across several research centers; some subjects from the same center, it may produce similarity or homogeneity. The methods that will be considered for adjusting for effects of the center will include the Breslow-Day method, Cochran-Mantel–Haenszel statistics (CMH), and logistical regression, whereas GEE and GLMM will be used for data mining. In addition, confounding factors will be considered in all data analysis, it will include gender, age, and other lung diseases.

#### Data analysis of the main outcome measure

The main outcome measure is treatment effect, and this categorical variable includes improvement, stabilization, and deterioration. Stabilization and deterioration will be merged into a new variable, namely non-improvement. Hence, this three-level classification will comprise merged binary variables and include improvement and non-improvement. For this dichotomous variable, Breslow-Day method, CMH, GEE, and logistic regression will be utilized to compare curative effects between the two groups.

#### Data analysis of secondary efficacy indices

For each subject from the treatment and control groups, the quantitative indicators of respiratory function (TLC, VC, DLCO, FVC, FVC%, VT, IC, 6-min walking distance, and Borg dyspnea score) will be collected several times and GLMM will be performed to determine the effectiveness of therapeutic modalities in a continuous time frame (0, 3, 6, 9, and 12 months).

The treatment effect in TCM syndrome includes clinical cure, notable effect, effectiveness, and ineffective. The clinical cure, notable effect, and effectiveness will be merged into a new variable named “total effective”. Hence, the treatment effect in TCM syndrome will comprise two new dichotomous variables, the qualitative variables, namely, “total effective” and “ineffective,” and will be recorded in each subject when they consult a physician across the time points in the study (0, 3, 6, 9, and 12 months). Hence, GEE will be conducted to determine the efficacy of treatment between the two groups.

#### Data analysis of quality of life

The SCL-90, SF-36, SAS scale, and SDS scales will be used to measure the quality of life of subjects in both groups when each of the patients will visit a study center during the continuous time points of the study (0, 3, 6, 9, and 12 months). All data are quantitative and GLMM will be conducted to explore the differences in indices between the two groups.

## Discussion

The novel coronavirus pneumonia has been responsible for several severe infections and a large number of deaths globally, with mortality ranging from 3–10% across different countries [4]. Individuals over 60 years of age, especially those with underlying diseases are more prone to mortality [10]. The pathological features of patients with COVID-19 are very similar to those of patients with severe acute respiratory syndrome (SARS) and the middle-east respiratory syndrome (MERS) [38]. These patients exhibit pulmonary fibrosis accompanied by immunopathological reactions. SARS-COV-2 can induce severe cellular immune responses in the lungs, resulting in severe lung tissue damage. The clinical manifestations are interstitial pneumonia, pulmonary fibrosis, and other pathological changes in the lungs [21, 39]. Upon recovery from severe COVID-19, pulmonary fibrosis caused by COVID-19 is found to be a common, yet serious problem in, which significantly reduces the quality of life.

The currently available treatment modalities cannot effectively control the development of pulmonary fibrosis and prevent lung-function deterioration [21, 39]. Therefore, there is an urgent requirement for new drug therapies, and the use of antifibrotic drugs should be considered as these have been proven to improve pulmonary fibrosiss [21, 39]. Pirfenidone is an orally administered antifibrotic drug that inhibits the expression of inflammatory factors [23] and reduces the proliferation of fibroblasts and collagen synthesis [23]. Thus, it significantly delays a decline of FEV capacity, reduces mortality rate, and improves lung function,

Moreover, many new Chinese herbs (including single and compound prescriptions), which have been used to treat lung damage in ancient as well as recent times, and may prove to be efficacious at preventing the decline in lung function. However, to date, no single herb or TCM therapy has been identified in the world of medicine as an effective treatment approach for the management of pulmonary fibrosis. Thus, further studies in the field of anti-fibrosis drug discovery are well warranted.

Pulmonary fibrosis is a chronic inflammatory disease characterized by fibroblast proliferation and excessive accumulation of extracellular matrix [40]. Epithelial-mesenchymal transition is the pivotal origin of myofibroblasts that secrete extracellular matrix, resulting in the development of pulmonary fibrosis [40]. Additionally, several cytokines, inflammatory mediators, and genetic factors contribute to the progression of tissue fibrosis [41, 42]. It is known that certain TCM formulae are effective at treating tissue injury and fibrosis [43]. This treatment modality involves multiple ingredients, numerous targets, and complicated mechanisms, and may provide us with a wide possibility to discover new drugs. Huaxian jian is a classical prescription that has been widely used in the treatment of patients with pulmonary fibrosis since the 1960s [30]. Previous studies have shown that Huaxian jian may exert an antifibrotic effect by inhibiting the secretion of the inflammatory factor, TNF-α, as well as inhibiting activation of the NF-κB inflammatory signaling pathway [44].

Based on clinical epidemiology and evidence-based medicine, our current study will explore the efficacy of the combination of TCM and synthetic drugs in the treatment of COVID-19 related lung-function decline in a clinical setting. Thus, our study will help establish a scientific, objective, and multi-dimensional system to evaluate the clinical efficacy, including TCM syndromes and the quality of life of patients. Syndrome is the key link of syndrome differentiation and treatment in TCM. In this study, we adopted the mode of combination of modern medicine and TCM, namely "combination of disease and syndrome"[45, 46]. Lastly, based on this study, we expect to discover a new comprehensive treatment approach to effectively improve pulmonary fibrosis associated with SARS-CoV-2, which likely improves the quality of life of patients.

## Limitations of this study

Our study has several shortcomings that may need to be addressed. First, this is a multi-center randomized controlled trial conducted over a 12-month period. Therefore, the long-term outcome of the treatment and control groups may not be obtained in a short time. Second, the components of TCM are complex and diverse, and these components are used as a mixture. Hence, it is difficult to identify or separate single chemical compounds from the complex mixture of herbs that are used to treat pulmonary fibrosis. Additionally, the mechanism of TCM in the prevention and treatment of pulmonary fibrosis is complex, and the mechanism of the interaction between TCM and modern medicines remain unclear. Hence, a large-sample, longstanding, double-blind RCT may have to be conducted to determine the long-term outcomes for different treatment plans. In addition, the principal components responsible for activity will have to be identified and extracted from herbs and further developed, following which, their efficacy in treating pulmonary fibrosis should be examined in future RCTs.

## Data Availability

Data of the study can be freely available to mail SX-Z.

## Abbreviations

AIDS: Acquired Immune Deficiency Syndrome
ALAT: Latin American Thoracic Association
ANOVA: Analysis of variance
ATS: American Thoracic Society
COPD: Chronic obstructive pulmonary disease
COVID-19: Corona Virus Disease 2019
CMH: Cochran-Mantel–Haenszel statistics
CRC: Clinical research coordinator
CRF: Case report form
DLCO: Diffusion capacity for carbon monoxide
DLCO%: The predicted diffusion capacity for carbon monoxide
ECMO: Extracorporeal membrane oxygenation
EDC: Electronic data capture
ERS: European Respiratory Society
ERV: Expiratory reserve volume
FEV1: Forced expiratory volume in 1 s
FiO_2_: Oxygen inhalation concentration
FLV: Forced lung volume
FVC: Forced vital capacity
FVC: The predicted forced vital capacity
GEE: Generalized estimation equation
GLMM: Generalized linear mixed model
HIV: Human immunodeficiency virus
IC: Inspiratory capacity
ICU: Intensive care unit
IQR: Inter-quartile range
IRV: Inspiratory reserve volume
kg: Kilogram
MERS: Middle East respiratory syndrome
mg: Milligram
MMEF: Maximal middle expiratory flow
MVV: The maximum vital capacity
PaO_2_: Partial pressure of oxygen
PCR: Polymerase chain reaction. PEF: Peak expiratory flow
PH: Hydrogen ion concentration
RCT: Randomized clinical trial
RNA: Ribonucleic acid
RV: Rsidual volume
SaO_2_: Arterial blood oxygen saturation
SARS: Severe acute respiratory syndrome
SARS-CoV-2: Severe acute respiratory syndrome coronavirus 2
SAS: Self-rating anxiety scale
SCL-90: Symptom self-evaluation scale
SD: Standard error
SDS: Self-rating depression scale
SF-36: Item short form health survey
SGRQ: St George's respiratory questionnaire
SpO_2_: Oxygen saturation
TLC: Total vital capacity
TCM: Traditional Chinese medicine
VC: Vital capacity
VT: Tide volume
WHO: World Health Organization
